# Longitudinal Associations Between Adolescents’ Attachment Preferences for Parents and Peers and Their (Mal)Adjustment

**DOI:** 10.3390/bs16050696

**Published:** 2026-05-02

**Authors:** Tomotaka Umemura, Yu Xu, Lenka Lacinová

**Affiliations:** 1 Program of Psychology, Graduate School of Humanities and Social Sciences, Hiroshima University, 1-1-1 Kagamiyama, Higashi-Hiroshima 739-8524, Japan; dpsy.xu@gmail.com; 2 The Faculty of Psychology, Kobe Gakuin University, 518 Arise, Ikawadani-cho, Nishi-ku, Kobe 651-2180, Japan; 3 Psychology Research Institute, Faculty of Social Studies, Masaryk University, Joštova 10, 602 00 Brno, Czech Republic; lacinova@fss.muni.cz; 4 Department of Psychology, Faculty of Social Studies, Masaryk University, Joštova 10, 602 00 Brno, Czech Republic

**Keywords:** adolescence, attachment preferences, internalizing and externalizing problems, multiple-informant approach, positive and negative affect schedule

## Abstract

Some adolescents prefer parents as their attachment figures, while others prefer peers, such as romantic partners and friends. However, how these attachment preferences influence (mal)adjustment is unclear. This study aimed to examine the longitudinal associations between adolescents’ preferences for attachment figures and their (mal)adjustment. We recruited 215 Czech adolescents (*M*_age_ = 14.02 in the 1st year, *SD* = 2.05, ranging between 11 and 18 years; girls = 54%) and utilized data from the adolescents’ reports of their attachment preferences in the 1st year of this project. In addition, adolescents, parents, and teachers reported adolescents’ (mal)adjustment over two years. The results showed that adolescents’ attachment preferences for mothers were longitudinally associated with lower parent-reported externalizing problems. On the other hand, attachment preferences for peers predicted lower teacher-reported internalizing problems. The findings suggest that attachment preferences for parents were linked to some more favorable adjustment outcomes, and that attachment preferences for peers may be more positively associated with adjustment when accompanied by attachment preferences for parents.

During adolescence…other adults may come to assume an importance equal to or greater than that of parents…At one extreme are adolescents who cut themselves off from parents; at the other are those who remain intensely attached and are unable or unwilling to direct their attachment behavior to others.

(Bowlby, 1969/1982, p. 207)

## 1. Introduction

Adolescence is a period during which many individuals show preference for parents and peers, such as friends and romantic partners, as attachment figures (Bowlby, 1969/1982; Hazan & Zeifman, 1994). However, whether these attachment preferences for parents and peers are associated with (mal)adjustment during adolescence remains unclear. Only a few cross-sectional empirical studies have demonstrated that the degree to which adolescents prefer their parents as their attachment figures had positive associations with their adjustment, while their preferences for peers as attachment figures had negative associations with their adjustment (Rosenthal & Kobak, 2010; Umemura et al., 2018; Xu & Umemura, 2025). To the best of our knowledge, no longitudinal study has been conducted with adolescents, and one longitudinal study in young adulthood found a positive association between preferences for peers and their adjustment (Mayseless, 2004). Hence, to examine whether adolescents’ attachment preferences are longitudinally related to their (mal)adjustment, we conducted a two-year longitudinal study. Data on adjustment were collected from multiple informants (adolescents, teachers, and parents).

### 1.1. Attachment Relationships as a Behavioral System

According to attachment theory (Bowlby, 1969/1982), a set of behaviors, referred to as the attachment behavioral system, functions to maintain proximity to a protective figure, especially under conditions of threat and distress. Although infants and children may direct attachment behaviors toward multiple caregivers, these figures are not treated as interchangeable. Instead, Bowlby (1969/1982, 1977) posited that individuals tend to seek a “differentiated and preferred individual” (Bowlby, 1977, p. 203). Based on this concept, Ainsworth (1989) proposed that children organize their multiple attachment relationships in a preferential order, hereafter called “attachment preferences.” In this preference structure, a primary figure (usually the mother) is preferentially sought for security, followed by subsidiary figures who function as attachment figures in reserve (Weiss, 1991; Colin, 1996). That is, when the primary figure is unavailable, individuals seek protection from other figures.

The distinction between attachment relationships and other supportive relationships, such as affiliation, has been carefully discussed. According to E. Waters and Cummings (2000), attachment and other supportive relationships can be effectively differentiated based on whether the situation is an emergency or a nonemergency. The affiliative behavioral system motivates individuals to seek company and shared interests, whereas the attachment system is specifically activated to seek security and protection (Crowell & Waters, 1994; Fraley & Shaver, 2000; Weiss, 1991).

Previous research has empirically demonstrated this distinction between attachment and other relationships. Rosenthal and Kobak (2010) developed a scale, the Important People Interview (IPI), which includes items that identify preferred figures during both emergency and non-emergency situations. They revealed that preferences in nonemergency contexts (e.g., seeking support for a “bad day”) may reflect affiliation or availability, whereas attachment figures are identified by preferences in high-threat emergency situations (e.g., regarding survival, like “hit by a car”). Evidence from non-Western samples (Japan and China) has further supported this construct differentiation, showing that attachment preferences are statistically distinct from affiliation (Umemura et al., 2022; Xu & Umemura, 2025). Therefore, in defining attachment preferences, this study focused specifically on “attachment relationships” for survival and security rather than general social support.

### 1.2. Longitudinal Associations Between Adolescents’ Attachment Preferences and (Mal)Adjustment

Adolescence is a major period for exhibiting attachment preferences for both parents and peers. For example, one of the first empirical studies on attachment preferences in school-aged children and adolescents (aged from 6 to 17 years) by Hazan and Zeifman (1994) demonstrated that most school-aged children and early and middle adolescents (aged younger than 15 years) reported that their parents provide a secure base, and they would feel desperate when separated from their parents. Nevertheless, approximately half of late adolescents (aged older than or equal to 15 years) perceived their peers as a secure base, and they would protest separating from them (see also Freeman & Brown, 2001; Nickerson & Nagle, 2005; Rowe & Carnelley, 2005; and Viejo et al., 2018 for cross-sectional findings; Friedlmeier & Granqvist, 2006 and Umemura et al., 2021 for longitudinal findings).

Furthermore, Kobak et al. (2007) hypothesized that a premature reorganization of attachment preferences would be a risk factor for adjustment problems. These premature relationships with peers who are perceived as important attachment figures are unlikely to be as stable and enduring as those that individuals develop later in adulthood. Specifically, the premature process of reorganization from parents to peers during early and middle adolescence is conceptually similar to premature autonomy from parents, which has been found to be associated with adjustment difficulties, such as delinquent or antisocial behaviors (Goldstein et al., 2005).

Associations between attachment preferences and (mal)adjustment have been examined mainly cross-sectionally, although evidence from longitudinal research is limited. Specifically, two Western cross-sectional studies from the U.S. and the Czech Republic (Rosenthal & Kobak, 2010; Umemura et al., 2018, who used the first year of this project) found associations of adolescents’ low preference for parents and high preference for peers with internalizing and externalizing behavior problems. The Czech study (Umemura et al., 2018) also found that attachment preferences for fathers were associated with higher positive affect, whereas attachment preferences for same-sex friends were related to higher negative affect. Moreover, consistent with Kobak et al.’s (2007) premature reorganization hypothesis, both attachment preferences for mothers and fathers were associated with low negative affect only for younger adolescents (not for older adolescents). Similarly, attachment preferences for fathers were related to high positive affect only for younger adolescents (not for older adolescents).

Importantly, two Asian cross-sectional studies have similarly demonstrated the association of adolescents’ low preferences for parents and high preferences for peers with higher adjustment problems. Specifically, Chinese adolescents who had high attachment preferences for mothers and fathers were more likely to have high self-esteem, low depressive symptoms, and low loneliness levels. Conversely, those who had high preferences for friends tended to experience these aspects of (mal)adjustment in the opposite direction (Xu & Umemura, 2025). Following the same pattern, Japanese university students who had high attachment preferences for mothers were more likely to have high self-esteem, and those who had high preferences for fathers were less likely to have low depressive symptoms (Umemura et al., 2022). Nevertheless, evidence regarding the longitudinal association between the attachment preferences and (mal)adjustment is lacking.

Interestingly, previous studies have found that *young adults*’ shift in attachment from parents to peers was either *positively* associated with their adjustment to a new environment (the study examined the adjustment to a military environment; Mayseless, 2004) or not associated with distress (a composite scale of two depression scales, anxiety, and physical symptoms; Pitman & Scharfe, 2010). These findings suggest that the role of attachment preferences in adjustment may differ across developmental periods.

Finally, these previous studies on attachment preferences (except for our previous study, Umemura et al., 2018) have relied solely on self-reported measures of adjustment, which is another limitation of the previous findings. The reliance on adolescent self-reports introduces the risk of shared method variance (Branstetter et al., 2009). Utilizing multi-informant data minimizes bias, facilitating a more comprehensive assessment of adolescent functioning.

### 1.3. The Present Study

To our knowledge, this study was the first to examine the longitudinal association between adolescents’ attachment preferences and their psychological (mal)adjustment, including externalizing and internalizing problems, as well as positive and negative affects. Other advantages of this study were that Bowlby’s (1969/1982) behavior systems were used to assess attachment preferences, and that (mal)adjustment was reported by multiple informants (parents, teachers, and adolescents).

Based on cross-sectional outcomes from previous studies and the theoretical framework of Kobak et al.’s (2007) premature attachment reorganization and its risks, we proposed the following hypotheses:

**Hypothesis** **1.** *Greater preferences for parents (mothers and fathers) would predict a high adjustment outcome (positive affect) and low maladjustment outcomes (negative affect, externalizing problems, and internalizing problems) compared to lower preferences for parents.*

**Hypothesis** **2.** *Higher preferences for peers (friends and romantic partners) would predict a low adjustment outcome (positive affect) and high maladjustment outcomes (negative affect and externalizing/internalizing problems) compared to lower preferences for peers.*

**Hypothesis** **3.** *The cohort would moderate the associations between attachment preferences and (mal)adjustment outcomes. Specifically, we expected that the protective effects of parental preferences and the risks of peer preferences would be stronger for younger adolescents than for older adolescents.*

## 2. Method

### 2.1. Participants

We recruited 215 Czech adolescents (*M*_age_ = 14.02, *SD* = 2.05, age range 11 to 18 years in the 1st year; girls = 54%) from specialized secondary schools that prepare students for higher education (called “gymnázium”) because our pilot study found that 11-year-old adolescents in “gymnázium” were able to understand the attachment preferences method that this study used, whereas early adolescents in other types of schools were not. For this reason, we believe that our participants represented an upper-middle-class sample. Ethical review and approval were waived for this study according to MU Directive No. 5/2015—Research Ethics at Masaryk University and Guidelines No. 3/2022 of the Faculty of Social Studies, Masaryk University, Guide to the Approval of Research Activities from the Perspective of Ethics. The recruited adolescents and their parents signed the informed consent form.

### 2.2. Procedure

Data were collected over 2 years, from October 2016 to June 2018. Adolescents reported attachment preferences and (mal)adjustment variables, including positive and negative affect. Their parents and teachers reported adolescents’ externalizing and internalizing problems. The present study used attachment preferences in the 1st year (October 2016) and (mal)adjustment variables in the 1st year (December 2016) and the 3rd year (June 2018). All adolescents and teachers completed paper-and-pencil questionnaires. Parents were initially asked to respond online. Those who did not respond online were given a paper-and-pencil form with a self-addressed stamped envelope.

### 2.3. Measures

**Attachment Preferences.** The IPI (Rosenthal & Kobak, 2010) consists of three distinct factors (*attachment bond, social support*, and *affiliation*), each measured by three items. This study used only the *attachment bond* factor that addresses Bowlby’s (1969/1982) attachment behavior systems. Participants were asked to rank attachment figures in four rows: the first row (5 points) was reserved for the primary attachment figure(s), the second row (4 points) was reserved for the next significant attachment figure(s), and so on (0 points for no nomination). We used a paper-and-pencil format (the original IPI was conducted online). Participants were instructed to assign the same rank to multiple attachment figures; e.g., peers and parents could be written in the same rank (see Umemura et al., 2018, for detailed information on our instructions). We calculated the median score for adolescents’ *mothers*, *fathers*, *friends*, and *romantic partners*. Cronbach’s alphas ranged from 0.74 (friends) to 0.99 (romantic partners).

**Positive and Negative Affect.** Adolescents also responded to a short version of the Positive and Negative Affect Schedule-Child Form (PANAS-C; Laurent et al., 1999). They were asked to rate the extent to which their affects during the last week reflect 5 positive and 5 negative affective words on a five-point Likert-type scale (1 = *It does not describe me, only nearly or at all*; 5 = *It absolutely describes me*). The internal consistencies for positive affect and negative affect ranged from α = 0.71 (negative affect in the 1st year) to α = 0.83 (positive affect in the 3rd year).

**Externalizing and Internalizing Problems.** We asked teachers and parents to complete the Strengths and Difficulties Questionnaire (SDQ; Goodman & Goodman, 2009), with each subcategory measured by 5 items on a three-point Likert-type scale (0 = *Not true*; 2 = *Certainly true*). Externalizing scores were computed by summing the scores for the *conduct problems* and *hyperactivity* subcategories. Internalizing scores were calculated by summing the *emotional problems* and *peer problems* subcategories. The internal consistencies ranged from α = 0.71 (parent-reported internalizing problems in the 1st year) to α = 0.85 (teacher-reported externalizing problems in the 1st year).

**Covariates.** This study included four cohorts: 21% were 5th graders (11 and 12 years in the 1st year), 34% were 7th graders (13 to 14 years in the 1st year), 27% were 9th graders (15 to 16 years in the 1st year), and 18% were 11th graders (17 to 18 years in the 1st year). Furthermore, 54% of adolescents were females. In our sample, 81.6% of adolescents lived with both biological parents. Finally, to determine the family SES, we calculated the composite score from the standardized scores for four SES variables: household income, SES score relative to other families in society, mother’s education, and father’s education (Cronbach’s α = 0.69). Concerning these SES variables individually, the mean household income was 2.80 (*SD* = 0.61) (*it is not enough to cover all expenses* = 1; *it tightly covers all expenses* = 2; *it covers all expenses without a problem* = 3; *it is high and we do not really worry about spending* = 4). The mean score for parents’ perception of their family’s SES relative to other families (*very poor* = 1; *very rich* = 10) was 5.91 (*SD* = 1.29). The average educational levels for mothers and fathers were 4.14 (*SD* = 1.06) and 4.09 (*SD* = 1.09), respectively (*a master degree or higher* = 5; *a bachelor or vocational higher-education degree* = 4; *a secondary school education with the final exam certification* = 3; *a secondary school education without the final exam* = 2; and *an elementary school education* = 1).

### 2.4. Analyses

We conducted multiple regression analyses to examine the effects of attachment preferences as an independent variable (IV) on psychological (mal)adjustment in the 3rd year as a dependent variable (DV). To better estimate changes in (mal)adjustment from the 1st to 3rd year, we included (mal)adjustment in the 1st year as an additional IV. Covariates (cohorts, sex, living arrangement, and family SES) were also added in the regression analyses. Additionally, we tested whether cohorts moderated these relationships. All analyses were performed in JASP (Version 0.19.3).

### 2.5. Missing Data Analyses

We found some missing data patterns. From the 1st to the 3rd year, 6% of adolescents, 20% of parents, and 4% of teachers were lost to follow-up. We conducted a series of Welch’s *t*-tests to compare the study variables collected in the 1st year between individuals who dropped out of this study in the 3rd year and those who did not. Adolescents who dropped out of this study were more likely to be older, *t*(16.50) = −6.36, *p* < 0.001, and less likely to live with both biological parents, *t*(11.70) *=* 2.76, *p* = 0.018, compared to those who completed it. Those adolescents also had lower attachment preferences for mothers, *t*(12.07) *=* 2.75, *p* = 0.018, and fathers, *t*(12.68) *=* 4.25, *p* < 0.001, and higher attachment preferences for romantic partners, *t*(11.35) = −2.61, *p* = 0.024. They also had higher parent-rated externalizing problems, *t*(10.41) = −2.29, *p* = 0.044, higher teacher-rated externalizing, *t*(11.93) = −2.86, *p* = 0.014, and higher teacher-rated internalizing problems, *t*(12.19) = −2.88, *p* = 0.014. Finally, parents and teachers who dropped out were more likely to have older adolescents compared to those who continued their participation, *t*(63.27) = −3.67, *p* < 0.001, and *t*(9.85) = −4.79, *p* < 0.001, respectively (see Table S1 for a complete set of *t*-test results). To address missing data, we employed Full Information Maximum Likelihood (FIML) estimation (interaction models with parent-rated externalizing and internalizing problems as outcomes were estimated pairwise because standard errors were otherwise not properly computed). Because attrition was related to several measured variables, the missingness pattern was unlikely to be completely random. Because missing not at random (MNAR) processes cannot be ruled out, our findings should be interpreted with caution (Baraldi & Enders, 2010).

## 3. Results

### 3.1. Descriptive Analyses

Table 1 presents the means and standard deviations for continuous variables, percentages for normative variables, and correlations between all study variables. Adolescents reported the highest attachment preferences for mothers, followed by fathers, and other family members. Friends ranked fourth, followed by romantic partners.

Adolescents who reported high preferences for parents (mothers and fathers) were likely to report low preferences for peers (friends and romantic partners). Younger cohorts had higher attachment preferences for mothers and fathers compared to older cohorts, whereas older cohorts had higher attachment preferences for friends and romantic partners. Girls were more likely than boys to report preferences for mothers. Finally, adolescents who lived with both biological parents showed higher attachment preferences for fathers than those who did not.

According to our correlation results between parent preferences and (mal)adjustment, higher attachment preferences for mothers were associated with high positive affect and low negative affect in the 3rd year. Similarly, higher attachment preferences for fathers were significantly associated with high positive affect in the 1st year and low negative affect in the 3rd year. Both attachment preferences for mothers and fathers had several significant associations with low externalizing and internalizing problems in the 1st and 3rd years.

Our correlations between attachment preferences for peers and (mal)adjustment revealed that those who reported higher attachment preferences for friends were more likely to have high negative affect in the 1st year. We did not find any significant associations for attachment preferences for romantic partners with (mal)adjustment.

### 3.2. Longitudinal Relations Between Attachment Preferences and (Mal)Adjustment

A series of multiple regression analyses were conducted to examine whether adolescents’ attachment preferences in the 1st year would predict (mal)adjustment in the 3rd year after controlling for (mal)adjustment in the 1st year, attachment preferences for other figures, cohort, sex, and family SES (see Table 2 for standardized coefficients and Table S2 for unstandardized coefficients with standard errors). Because six regression tests included four attachment figures (6 × 4 = 24 coefficients), we controlled the false discovery rate using the Benjamini–Hochberg procedure.

The results indicated that, after controlling for those covariates and the false discovery rate, greater attachment preferences for mothers in the 1st year were significantly associated with low parent-reported externalizing problems in the 3rd year. Contrary to the direction predicted by our hypothesis, attachment preferences for friends in the 1st year were significantly associated with *low* teacher-reported internalizing problems in the 3rd year. We did not find any significant associations regarding attachment preferences for fathers and romantic partners. Sensitivity analyses were also conducted with the complete data cases, and results did not differ from the main results (see Table S4).

To examine the interaction effect of attachment preferences and cohorts on (mal)adjustment, we independently conducted additional regression analyses. Specifically, we added an interaction variable multiplied by attachment preferences and cohort to the previous regression models. We again controlled the false discovery rate using the Benjamini–Hochberg procedure for 24 coefficients (six regression tests × four attachment figures. The results indicated no significant interaction effects (see Table 3 for standardized results and Table S3 for unstandardized results).

## 4. Discussion

Numerous studies have been conducted on attachment from infancy through adolescence to adulthood. However, how adolescents organize their attachment preferences for their attachment figures, such as parents and peers, and whether their preferences for attachment figures are associated with (mal)adjustment, remain unclear. This study focused on the latter question and contributed to the existing literature by examining the longitudinal associations between adolescents’ attachment preferences and their (mal)adjustment. In addition, as advantages of this study, we used methodologically rigorous approaches, employing Bowlby’s (1969/1982) idea of attachment behavior systems and assessing (mal)adjustment as reported by parents, teachers, and adolescents themselves. Overall, the pattern of our findings was mixed and informant-specific.

### 4.1. Attachment Preferences and (Mal)Adjustment

Consistent with our *Hypothesis 1*, adolescents’ attachment preferences for parents were longitudinally related to their low maladjustment. Specifically, regardless of adolescents’ development (across cohorts), other attachment figures, and other covariates, high attachment preferences for mothers were longitudinally related to low parent-reported externalizing problems. This finding was also in line with the findings from previous cross-sectional studies (Rosenthal & Kobak, 2010; Umemura et al., 2022; Xu & Umemura, 2025). In general, the cross-sectional and longitudinal findings suggest that attachment preferences for parents are protective factors against adolescents’ adjustment problems. For example, consider a scenario in which adolescents encounter negative experiences at school. Those who seek security from their parents by discussing these difficulties can benefit from receiving appropriate advice and emotional support.

Conversely, inconsistent with our *Hypothesis 2*, after controlling for the effects of cohorts, other attachment figures, and covariates, high attachment preferences for peers, specifically friends, predicted low teacher-reported internalizing problems. Internalizing problems were composed of the sum of the emotional problem and peer problem subcategories. Teachers rate peer problems based on their observations of adolescents in schools. Thus, attachment preferences for friends may function well in adolescents’ peer relationships in school contexts. Thus, attachment preferences for peers may be adaptive in some contexts. Previous studies using the IPI have not used teacher-reported (mal)adjustment but only self-reported data (e.g., Rosenthal & Kobak, 2010; Xu & Umemura, 2025), except for one study that used the cross-sectional data of this study (Umemura et al., 2018). Therefore, previous studies might not be able to accurately evaluate school contexts.

In addition, when carefully looking at the correlation results, attachment preferences for friends were related only to concurrent negative affect and not to other (mal)adjustment variables, suggesting that attachment preferences for friends showed a more limited pattern of associations with (mal)adjustment than attachment preferences for parents. This pattern of results was also consistent with Japanese findings (Umemura et al., 2022), in which only parental attachment was significantly associated with mal(adjustment), while peer attachment was not.

Contrary to our *Hypothesis 3*, we did not find any interaction effects between attachment preferences and cohorts on (mal)adjustment. Initially, we anticipated that the protective effects of parental preferences and the risks of peer preferences would be more pronounced in younger adolescents compared to older adolescents. However, the results derived from this longitudinal study were inconsistent with our expectations, as well as Kobak et al.’s (2007) hypothesis and our earlier cross-sectional results (Umemura et al., 2018). That is, Kobak et al. (2007) hypothesized that early reorganization of attachment to peers would lead to delinquent or antisocial behaviors. Our earlier cross-sectional results supported Kobak’s hypothesis (Umemura et al., 2018). We interpret that although a low level of attachment preferences for parents may increase the risk for adolescents’ (mal)adjustment in early adolescence, it remains a risk factor throughout the entire adolescence period. Some evidence from Asian cross-sectional data also suggests that a low level of attachment preferences for parents in Japanese college students is associated with (mal)adjustment (Umemura et al., 2022).

According to the findings from our longitudinal study, along with those from previous studies, attachment preferences for parents may be associated with more favorable adjustment during adolescence. However, individual circumstances need to be considered as well. For example, children and adolescents who have experienced physical, sexual, or psychological abuse may be encouraged to change their attachment preferences from parents to other people. Moreover, for those whose parents or other stable caregivers are temporarily unavailable, peers may be particularly important sources of emotional security. Thus, additional longitudinal research across diverse individuals and societies is essential before drawing broader practical implications or disseminating a straightforward message to the public.

### 4.2. Limitations

Studies on adolescents’ and young adults’ attachment preferences have mostly relied on self-reports, regardless of the methods used, e.g., interviews, open-ended questions, and Likert-type scales. Consequently, some young individuals may provide responses that align with cultural norms, which may result in an inaccurate self-assessment. New assessments of attachment preferences should be developed by drawing upon ideas from existing attachment assessments, such as the adult attachment interview (Main et al., 2003) or the attachment script assessment (H. S. Waters & Waters, 2006).

Finally, the participants in this study were recruited from specialized secondary schools that prepare students for higher education (called “gymnázium”) in the Czech Republic. Therefore, the generalizability of our findings to students in other educational tracks, such as vocational schools, and to countries other than the Czech Republic should be considered carefully.

## 5. Conclusions

During adolescence, some adolescents prefer parents as their attachment figures, while others prefer peers. This study revealed that adolescents’ higher attachment preferences for mothers were longitudinally related to lower parent-reported externalizing problems. This finding, along with those from previous studies, suggests that attachment preferences for parents may be linked to some more favorable adjustment outcomes during adolescence. This study also found that higher attachment preferences for friends were longitudinally related to lower teacher-reported internalizing problems. The developmental process of transition from parent–child attachment to adult peer attachment is still unclear, and the research on this topic is limited. In addition, our results were mixed and informant-specific. Thus, more studies are needed to understand normative patterns and individual differences in attachment preferences.

## Figures and Tables

**Table 1:** Means (SDs), percentages, and correlation coefficients among study variables.

		*M* or *%*	(*SD*)	1	2	3	4	5	6	7	8	9	10	11	12	13	14	15	16	17	18	19
Attachment preferences for																						
1	Mothers	3.29	(1.34)	-	-	-	-	-	-	-	-	-	-	-	-	-	-	-	-	-	-	-
2	Fathers	2.52	(1.60)	0.47 ***	-	-	-	-	-	-	-	-	-	-	-	-	-	-	-	-	-	-
3	Friends	1.51	(1.55)	−0.29 ***	−0.29 ***	-	-	-	-	-	-	-	-	-	-	-	-	-	-	-	-	-
4	Romantic partners	0.16	(0.54)	−0.28 ***	−0.24 ***	0.08	-	-	-	-	-	-	-	-	-	-	-	-	-	-	-	-
Positive and negative affects (rated by adolescents)																						
5	Positive affect in Year 1	3.26	(0.77)	0.08	0.15 *	−0.10	−0.05	-	-	-	-	-	-	-	-	-	-	-	-	-	-	-
6	Negative affect in Year 1	2.16	(0.78)	−0.08	−0.07	0.15 *	−0.05	−0.38 ***	-	-	-	-	-	-	-	-	-	-	-	-	-	-
7	Positive affect in Year 3	3.28	(0.86)	0.15 *	0.12	−0.01	0.08	0.45 ***	−0.13	-	-	-	-	-	-	-	-	-	-	-	-	-
8	Negative affect in Year 3	2.32	(0.94)	−0.19 **	−0.15 *	0.11	−0.05	−0.17 *	0.36 ***	−0.54 ***	-	-	-	-	-	-	-	-	-	-	-	-
Externalizing and internalizing problems (rated by parents)																						
9	Externalizing problems in Year 1	3.48	(3.06)	−0.09	−0.24 ***	0.14	0.04	−0.07	0.06	−0.05	0.10	-	-	-	-	-	-	-	-	-	-	-
10	Internalizing problems in Year 1	4.31	(3.10)	−0.00	−0.16 *	0.03	−0.01	−0.15 *	0.23 **	−0.25 ***	0.21 **	0.26 ***	-	-	-	-	-	-	-	-	-	-
11	Externalizing problems in Year 3	2.94	(2.60)	−0.15 *	−0.21 **	0.09	−0.01	−0.12	0.07	−0.07	0.02	0.71 ***	0.17 *	-	-	-	-	-	-	-	-	-
12	Internalizing problems in Year 3	3.69	(2.88)	−0.02	−0.18 *	−0.05	−0.05	−0.18 *	0.21 **	−0.24 **	0.24 **	0.31 ***	0.70 ***	0.28 ***	-	-	-	-	-	-	-	-
(rated by teachers)																						
13	Externalizing problems in Year 1	2.81	(3.29)	−0.27 ***	−0.16 *	0.05	−0.01	−0.02	0.09	0.00	0.14	0.33 ***	−0.08	0.32 ***	−0.01	-	-	-	-	-	-	-
14	Internalizing problems in Year 1	3.82	(3.11)	−0.17 *	−0.36 ***	0.01	0.06	0.03	0.07	−0.15 *	0.12	0.20 **	0.31 ***	0.16 *	0.36 ***	0.19 **	-	-	-	-	-	-
15	Externalizing problems in Year 3	3.16	(3.12)	−0.17 *	−0.13	0.01	−0.05	−0.03	0.03	0.02	0.13	0.35 ***	−0.10	0.42 ***	0.03	0.76 ***	0.17 *	-	-	-	-	-
16	Internalizing problems in Year 3	3.87	(2.91)	−0.12	−0.33 ***	−0.09	0.08	0.02	0.10	−0.15 *	0.20 **	0.14 *	0.33 ***	0.09	0.34 ***	0.06	0.68 ***	0.11	-	-	-	-
Covariates																						
17	Cohorts	2.43	(1.02)	−0.26 ***	−0.39 ***	0.27 ***	0.40 ***	−0.10	0.18 **	−0.06	0.20 **	0.14 *	0.06	−0.07	−0.02	0.16 *	0.23 ***	0.03	0.30 ***	-	-	-
18	Sex (girls)	54.0%		0.18 *	0.03	0.12	0.08	0.02	0.21 **	0.02	0.07	−0.07	0.10	−0.17 *	0.06	−0.31 ***	−0.10	−0.28 ***	−0.09	−0.02	-	-
19	Living arrangement	81.3%		0.01	0.30 ***	0.01	0.01	−0.06	0.03	−0.06	0.01	−0.11	0.01	−0.07	0.01	−0.12	−0.10	−0.10	−0.13	−0.24 ***	−0.15 *	-
20	Family SES	0.00	(0.74)	0.05	0.11	−0.09	0.00	0.01	−0.01	0.08	−0.14	−0.22 **	−0.16 *	−0.01	−0.11	−0.08	−0.05	−0.10	−0.06	−0.12	0.11	0.15 *

*Note.* Cohorts (1 = 5th graders; 2 = 7th graders; 3 = 9th graders; 4 = 11th graders). Sex (0 = boys; 1 = girls). Living arrangement (1 = adolescents lived with both biological parents; 0 = adolescents did not). *** *p* < 0.001. ** *p* < 0.01. * *p* < 0.05.

**Table 2:** Standardized regression coefficients of attachment preferences and covariates on (mal)adjustment.

	Self-Reported Positive Affect in Year 3			Self-Reported Negative Affect in Year 3			P-Rated Externalizing Problems in Year 3			P-Rated Internalizing Problems in Year 3			T-Rated Externalizing Problems in Year 3			T-Rated Internalizing Problems in Year 3		
	β	*p*	*q* ^(BH)^	β	*p*	*q* ^(BH)^	β	*p*	*q* ^(BH)^	β	*p*	*q* ^(BH)^	β	*p*	*q* ^(BH)^	β	*p*	*q* ^(BH)^
AP for mothers	0.16	0.041		−0.16	0.041		−0.16	0.004	*	−0.09	0.162		0.04	0.490		0.02	0.791	
AP for fathers	0.05	0.518		−0.05	0.565		−0.02	0.723		−0.11	0.118		−0.12	0.025		−0.14	0.042	
AP for friends	0.09	0.189		−0.04	0.605		0.08	0.133		−0.08	0.179		0.00	0.926		−0.18	0.001	*
AP for partners	0.15	0.042		−0.15	0.063		0.02	0.710		0.00	0.980		0.02	0.677		−0.05	0.381	
Cohort	−0.03	0.741		0.15	0.058		−0.23	<0.001		−0.08	0.238		−0.13	0.011		0.16	0.009	
Female (vs. male)	−0.03	0.696		0.04	0.605		−0.07	0.195		0.04	0.514		−0.07	0.157		−0.01	0.793	
Living arrangement	−0.07	0.336		0.08	0.289		−0.03	0.601		−0.02	0.704		0.04	0.378		0.01	0.924	
Family SES	0.08	0.245		−0.13	0.060		0.12	0.014		0.02	0.776		−0.09	0.033		−0.04	0.417	
(Mal)adjustment in Year 1	0.43	<0.001		0.29	<0.001		0.76	<0.001		0.71	<0.001		0.78	<0.001		0.60	<0.001	

*Note.* T = teacher. P = parent. AP = attachment preferences. *p* = unadjusted *p* value; *q*^(BH)^ = Benjamini–Hochberg adjusted *p* value. * = Statistically significant after Benjamini–Hochberg adjustment for multiple testing.

**Table 3:** Standardized regression coefficients with interaction effects added.

	Self-Reported Positive Affect in Year 3			Self-Reported Negative Affect in Year 3			P-Rated Externalizing Problems in Year 3			P-Rated Internalizing Problems in Year 3			T-Rated Externalizing Problems in Year 3			T-Rated Internalizing Problems in Year 3		
	β	*p*	*q* ^(BH)^	β	*p*	*q* ^(BH)^	β	*p*	*q* ^(BH)^	β	*p*	*q* ^(BH)^	β	*p*	*q* ^(BH)^	β	*p*	*q* ^(BH)^
AP for mothers	0.15	0.061		−0.15	0.079		−0.09	0.180		−0.05	0.961		0.07	0.219		0.06	0.331	
AP for fathers	0.02	0.839		−0.03	0.704		−0.10	0.112		−0.13	0.961		−0.14	0.008		−0.14	0.034	
AP for friends	0.10	0.165		−0.04	0.608		0.05	0.392		−0.07	0.961		0.01	0.911		−0.17	0.003	
AP for partners	0.28	0.094		−0.32	0.064		−0.06	0.594		−0.14	1.000		0.10	0.371		−0.15	0.283	
Cohort	−0.33	0.178		0.35	0.167		−0.25	0.195		0.14	0.0961		−0.02	0.894		0.52	0.008	
Female (vs. male)	−0.03	0.692		0.03	0.700		−0.08	0.149		0.03	0.961		−0.07	0.147		−0.02	0.702	
Living arrangement	−0.05	0.484		0.08	0.335		−0.03	0.634		0.00	0.977		0.06	0.244		0.02	0.732	
Family SES	0.05	0.415		−0.12	0.080		0.11	0.036		0.01	0.963		−0.10	0.028		−0.04	0.465	
(Mal)adjustment in Year 1	0.47	<0.001		0.33	<0.001		0.68	<0.001		0.72	0.961		0.79	<0.001		0.60	<0.001	
AP for mothers × Cohort	−0.10	0.676		0.03	0.910		−0.06	0.726		−0.30	0.961		−0.23	0.124		−0.26	0.159	
AP for fathers × Cohort	0.38	0.013		−0.21	0.184		0.12	0.300		0.14	0.961		0.16	0.115		−0.05	0.689	
AP for friends × Cohort	0.07	0.440		−0.07	0.472		−0.07	0.317		−0.09	0.961		−0.03	0.621		−0.10	0.182	
AP for partners × Cohort	−0.09	0.578		0.17	0.335		0.11	0.362		0.14	1.000		−0.08	0.470		0.07	0.594	

*Note.* T = teacher. P = parent. AP = attachment preferences. Cohort were centered to avoid a multicollinearity problem. *p* = unadjusted *p* value; *q*^(BH)^ = Benjamini–Hochberg adjusted *p* value. No statistically significant results were found after Benjamini–Hochberg adjustment for multiple testing.

## Data Availability

The data presented in this study are openly available at https://osf.io/gvh8t (accessed on 27 April 2026).

## Supplementary Materials

The following supporting information can be downloaded at: https://www.mdpi.com/article/10.3390/bs16050696/s1, Table S1: T-Test Analyses for Missing data; Table S2: Unstandardized Regression Coefficients (Standard Errors) of Attachment Preferences and Covariates on (Mal)adjustment; Table S3: Unstandardized Regression Coefficients (Standard Errors) Adding Interaction Effects; Table S4: Standardized Regression Coefficients (Complete Case Analyses).
