# Supplementary material for: Longitudinal Associations Between Adolescents’ Attachment Preferences for Parents and Peers and Their (Mal)Adjustment
Source: Behav Sci (Basel). 2026 May 2;16(5):696. doi: 10.3390/bs16050696 (PMC13203552; doi:10.3390/bs16050696)
Supplement: Supplementary file 1 [file behavsci-16-00696-s001.zip › behavsci-4172656-supplementary.pdf]

Table S1.

T-Test Analyses for Missing data.

	Continued adolescents		Discontinued adolescents					Continued parents		Discontinued parents					Continued teachers		Discontinued teachers				
	Mean	*SD*	*Mean*	*SD*	*t*	*df*	*p*	*Mean*	*SD*	*Mean*	*SD*	*t*	*df*	*p*	*Mean*	*SD*	*Mean*	*SD*	*t*	*df*	*p*
Cohort	2.37	1.01	3.42	0.52	-6.36	16.50	< .001	2.30	0.98	2.93	1.01	-3.67	63.27	< .001	2.40	1.02	3.25	0.46	-4.79	9.85	< .001
Sex	0.45	0.50	0.58	0.52	-0.85	12.25	0.41	0.47	0.50	0.44	0.50	0.27	64.42	0.79	0.46	0.50	0.50	0.54	-0.21	7.48	0.84
Family SES	0.01	0.74	-0.11	0.79	0.46	9.84	0.65	0.02	0.74	-0.09	0.75	0.77	44.99	0.44	0.01	0.74	-0.26	0.78	0.92	6.40	0.39
Living Arrangement	0.83	0.37	0.42	0.52	2.76	11.70	0.02	0.82	0.39	0.77	0.43	0.73	60.21	0.47	0.82	0.38	0.50	0.54	1.68	7.28	0.14
AP for mothers	3.36	1.30	2.17	1.47	2.75	12.07	0.02	3.35	1.31	3.05	1.45	1.26	60.65	0.21	3.34	1.31	2.13	1.64	2.06	7.35	0.08
AP for fathers	2.62	1.55	0.83	1.40	4.25	12.68	< .001	2.61	1.56	2.16	1.72	1.54	60.82	0.13	2.57	1.57	1.13	1.81	2.23	7.42	0.06
AP for friends	1.49	1.53	1.83	1.80	-0.64	11.98	0.53	1.49	1.53	1.60	1.62	-0.43	62.59	0.67	1.49	1.54	2.13	1.81	-0.98	7.40	0.36
AP for romantic partners	0.12	0.48	0.83	0.94	-2.61	11.35	0.02	0.13	0.48	0.30	0.71	-1.55	52.25	0.13	0.14	0.51	0.63	0.92	-1.48	7.17	0.18
Self-report positive affect	3.26	0.75	3.22	1.07	0.15	11.65	0.89	3.25	0.73	3.29	0.91	-0.25	56.47	0.80	3.26	0.74	3.25	1.26	0.03	7.19	0.98
Self-report negative affect	2.15	0.77	2.23	0.99	-0.27	11.81	0.79	2.11	0.77	2.36	0.80	-1.86	63.30	0.07	2.15	0.77	2.38	1.12	-0.56	7.26	0.59
P-rated externalizing	3.30	2.85	6.64	4.80	-2.29	10.41	0.04	3.28	2.88	4.41	3.69	-1.73	44.58	0.09	3.37	2.91	6.43	5.44	-1.48	6.12	0.19
P-rated internalizing	4.20	3.07	6.18	3.22	-1.99	11.06	0.07	4.23	3.01	4.67	3.54	-0.70	46.42	0.49	4.21	3.05	7.00	3.70	-1.97	6.29	0.09
T-rated externalizing	2.63	3.18	5.83	3.81	-2.86	11.93	0.01	2.57	3.12	3.74	3.82	-1.87	56.79	0.07	2.71	3.24	5.25	4.03	-1.76	7.36	0.12
T-rated internalizing	3.66	3.04	6.42	3.23	-2.88	12.19	0.01	3.62	3.03	4.61	3.35	-1.76	60.41	0.08	3.72	3.05	6.25	3.77	-1.87	7.36	0.10

Note. T = teacher. P = parent. AP = attachment preferences. Cohorts (1 = 5th graders; 2 = 7th graders; 3 = 9th graders; 4 = 11th graders). Sex (0 = boys; 1 = girls). Living arrangement (1 = adolescents lived with both biological parents; 0 = those did not).

Table S2.

Unstandardized Regression Coefficients (Standard Errors) of Attachment Preferences and Covariates on (Mal)adjustment.

	Self-reported positive affect in Year 3	Self-reported negative affect in Year 3	P-rated externalizing problems in Year 3	P-rated internalizing problems in Year 3	T-rated externalizing problems in Year 3	T-rated internalizing problems in Year 3
AP for mothers	.10(.05)	-.11(.06)	-.33(.12)	-.20(.14)	.09(.12)	.04(.13)
AP for fathers	.03(.04)	-.03(.05)	-.04(.10)	-.20(.13)	-.24(.11)	-.24(.12)
AP for friends	.05(.03)	-.02(.04)	.14(.09)	-.15(.11)	-.01(.10)	-.33(.10)
AP for partners	.26(.13)	-.27(.15)	.11(.31)	.01(.37)	.13(.30)	-.29(.33)
Cohort	-.02(.07)	.14(.08)	-.63(.17)	-.23(.20)	-.41(.16)	.45(.18)
Female (vs. male)	-.05(.12)	.07(.14)	-.37(.28)	.22(.34)	-.43(.30)	-.08(.32)
Living arrangement	-.16(.17)	.20(.19)	-.20(.38)	-.18(.46)	.35(.39)	.04(.43)
Family SES	.09(.08)	-.16(.09)	.45(.18)	.06(.22)	-.40(.19)	-.16(.20)
(Mal)adjustment in Year 1	.48(.08)	.35(.08)	.66(.05)	.67(.05)	.74(.04)	.56(.05)

Note. T = teacher. P = parent. AP = attachment preferences.

*** $p < .001$. ** $p < .01$. * $p < .05$.

Table S3.

Unstandardized Regression Coefficients (Standard Errors) Adding Interaction Effects.

	Self-reported positive affect in Year 3	Self-reported negative affect in Year 3	P-rated externalizing problems in Year 3	P-rated internalizing problems in Year 3	T-rated externalizing problems in Year 3	T-rated internalizing problems in Year 3
AP for mothers	.10(.05)	-.11(.06)	-.17(.13)	.06(.14)	.16(.13)	.14(.14)
AP for fathers	.01(.05)	-.02(.05)	-.16(.10)	-.28(.12)	-.28(.11)	-.25(.12)
AP for friends	.05(.04)	-.02(.04)	.08(.09)	-.13(.11)	.01(.10)	-.31(.11)
AP for partners	.47(.28)	-.59(.32)	-.32(.60)	-.83(.69)	.62(.70)	-.81(.76)
Cohort	-.29(.22)	.34(.24)	-.66(.51)	.41(.59)	-.07(.53)	1.51(.58)
Female (vs. male)	-.05(.12)	.05(.14)	-.40(.28)	.15(.32)	-.43(.30)	-.12(.32)
Living arrangement	-.12(.17)	.18(.19)	-.18(.37)	.12(.42)	.46(.39)	.15(.43)
Family SES	.06(.08)	-.15(.09)	.38(.18)	.00(.21)	-.41(.19)	-.15(.20)
(Mal)adjustment in Year 1	.53(.08)	.39(.09)	.57(.04)	.62(.05)	.74(.04)	.57(.05)
AP for mothers × Cohort	-.02(.06)	.01(.06)	-.05(.13)	-.29(.15)	-.21(.14)	-.21(.15)
AP for fathers × Cohort	.11(.05)	-.07(.05)	.11(.10)	.21(.12)	.17(.11)	-.05(.12)
AP for friends × Cohort	.03(.04)	-.03(.05)	-.09(.09)	-.17(.11)	-.05(.10)	-.14(.11)
AP for partners × Cohort	-.11(.20)	.22(.23)	.40(.44)	.47(.50)	-.36(.49)	.29(.54)

Note. T = teacher. P = parent. AP = attachment preferences. Cohort were centered to avoid a multicollinearity problem.
*** $p < .001$. ** $p < .01$. * $p < .05$.

Table S4.

Standardized Regression Coefficients (Complete case Analyses).

	Self-reported positive affect in Year 3			Self-reported negative affect in Year 3			P-rated externalizing problems in Year 3			P-rated externalizing problems in Year 3			T-rated externalizing problems in Year 3			T-rated internalizing problems in Year 3		
	β	*p*	$q^{(BH)}$	β	*p*	$q^{(BH)}$	β	*p*	$q^{(BH)}$	β	*p*	$q^{(BH)}$	β	*p*	$q^{(BH)}$	β	*p*	$q^{(BH)}$
AP for mothers	.15	.041		-.16	.041		-.17	.004	*	-.09	.164		.04	.490		.02	.791	
AP for fathers	.05	.518		-.05	.565		-.02	.722		-.11	.117		-.12	.025		-.14	.042	
AP for friends	.09	.188		-.04	.605		.08	.134		-.08	.179		.00	.926		-.18	.001	*
AP for partners	.15	.041		-.14	.063		.02	.711		.00	.980		.02	.677		-.05	.381	
Cohort	-.03	.741		.15	.058		-.24	< .001		-.08	.238		-.13	.011		.16	.009	
Female (vs. male)	-.03	.696		.04	.605		-.07	.194		.04	.514		-.07	.157		-.01	.793	
Living arrangement	-.07	.337		.08	.289		-.03	.601		-.02	.704		.04	.378		.01	.924	
Family SES	.08	.245		-.13	.060		.13	.014		.02	.776		-.09	.034		-.04	.418	
(Mal)adjustment in Year 1	.42	< .001		.29	< .001		.74	< .001		.70	< .001		.78	< .001		.59	< .001	

Note. T = teacher. P = parent. AP = attachment preferences.

*** $p < .001$. ** $p < .01$. * $p < .05$.
